# Orthopedic Risks and Recovery Considerations in Long COVID: A Narrative Review

**DOI:** 10.7759/cureus.116021

**Published:** 2026-09-09

**Authors:** Kyle N Zelenski, Gavin K Christensen, Jeremy Hart, Jordan C Speth, Zachary M Harris, Kiefer J Zelenski, Bhavya Gupta, Temi D Ogunleye

**Affiliations:** 1 College of Osteopathic Medicine, Des Moines University, West Des Moines, USA; 2 Department of Biological Sciences, California State University, Chico, Chico, USA; 3 Department of Orthopedic Surgery, St. Joseph's Medical Center, Stockton, USA

**Keywords:** bone mineral density, chronic pain, covid-19, long covid, orthopedic surgery, perioperative complications, rehabilitation, wound healing

## Abstract

The COVID-19 pandemic left a lasting imprint on orthopedic surgery, initially through widespread reductions in care delivery and subsequently through the complex challenges posed by Long COVID syndrome. Emerging literature describes Long COVID-related alterations in bone mineral density, delayed wound healing, prolonged inflammation, persistent chronic pain, and increased perioperative risks. Long COVID may adversely affect skeletal integrity through inflammatory, hypoxic, and angiotensin-converting enzyme 2-mediated pathways, potentially leading to bone loss and increased fracture susceptibility. Persistent inflammation, endothelial dysfunction, thrombotic tendency, and neurocognitive impairment may also increase perioperative vulnerability, while chronic musculoskeletal pain, reduced exercise capacity, and post-exertional malaise may complicate rehabilitation and functional recovery. Although orthopedic-specific evidence remains limited, the available findings suggest that Long COVID may represent an underrecognized risk modifier relevant to surgical planning and postoperative management. Greater awareness of these mechanisms may improve patient counseling, risk stratification, and rehabilitation strategies.

## Introduction and background

In late 2019, a novel virus emerged in Wuhan, China. Genetic analysis revealed it to be closely related to the severe acute respiratory syndrome coronavirus, leading to its designation as severe acute respiratory syndrome coronavirus 2 (SARS-CoV-2). Within the first three months of 2020, the virus spread rapidly worldwide, prompting the World Health Organization (WHO) to declare a global pandemic [1]. SARS-CoV-2 causes the coronavirus disease 2019 (COVID-19), which has led to profound loss of life, widespread mental health consequences, and severe economic disruptions. Healthcare systems were particularly affected, as growing awareness of the COVID-19 threat compelled many patients to delay seeking care. At the same time, hospital resources were diverted to treating COVID-19 patients, leading to delays and cancellations of other medical services, including elective surgeries [2-4].

As early as spring 2020, patients who did not recover from COVID-19 within the expected two-week period began to experience a condition known as "Long COVID" [5]. Also referred to as post-COVID-19 syndrome or post-acute sequelae of COVID-19, this condition encompasses a spectrum of symptoms that persist for weeks to months, often defined as lasting beyond 12 weeks after infection [6]. Concerningly, in a 2025 meta-analysis of 429 studies, Hou et al. [7] reported a pooled global Long COVID prevalence of 36% (95% CI, 33-40%) across 144 contributing studies among individuals with a confirmed COVID-19 diagnosis. Notably, when stratified by follow-up duration across studies, Long COVID prevalence did not decline over time; rather, it increased from 35% (95% CI, 31-39%) within the first year of follow-up to 46% (95% CI, 37-57%) at one to two years. Hospitalized patients had a higher pooled prevalence (44%) than non-hospitalized patients (29%), and female sex emerged as one of the strongest demographic risk factors (pooled OR 1.55, 95% CI 1.25-1.92) [7]. Commonly reported manifestations include fatigue, fever, respiratory distress, cognitive impairment ("brain fog"), paresthesia, gastrointestinal issues, joint pain, and muscle pain [8].

The emergence of Long COVID has introduced new challenges for orthopedic care. The strongest current evidence concerns persistent musculoskeletal (MSK) symptoms and functional impairment [9], yet the broader implications for orthopedic practice remain poorly defined. To our knowledge, this is the first review to synthesize these findings across the continuum of orthopedic care. This review examines how Long COVID may influence MSK care, perioperative risk, and recovery trajectories, and identifies where further research is needed to guide orthopedic practice.

Methodology

A comprehensive literature search was conducted to identify relevant articles that investigated and synthesized the impact of Long COVID on orthopedic care in the post-pandemic period. Electronic databases, including PubMed/MEDLINE, Scopus, and Google Scholar, were queried through July 2026. Searches used variations of "Long COVID," "post-acute sequelae of SARS-CoV-2," and "PASC," with one or more of the following key terms: "orthopedic surgery," "orthopedic care," "musculoskeletal care," "bone mineral density," "fracture," "wound healing," "inflammatory response," "chronic pain," "rehabilitation," "perioperative risk," and "postoperative complications." MeSH terms identified in PubMed/MEDLINE were reused as additional keywords in Scopus and Google Scholar searches.

Given the emerging and heterogeneous nature of the Long COVID literature, broad inclusion criteria were applied. Eligible sources included original research, case series, case reports, and review articles published in English. Due to the limited number of articles directly investigating orthopedic populations, findings were synthesized narratively, and a formal risk-of-bias assessment was not performed.

## Review

Clinically, Long COVID has been associated with reduced bone mineral density (BMD), delayed wound healing, avascular necrosis (AVN), chronic inflammatory responses, MSK pain syndromes, and perioperative vulnerability (Figure 1) [9-15]. Each of these may influence orthopedic decision-making across the preoperative, operative, and postoperative phases of care.

**Figure 1:**
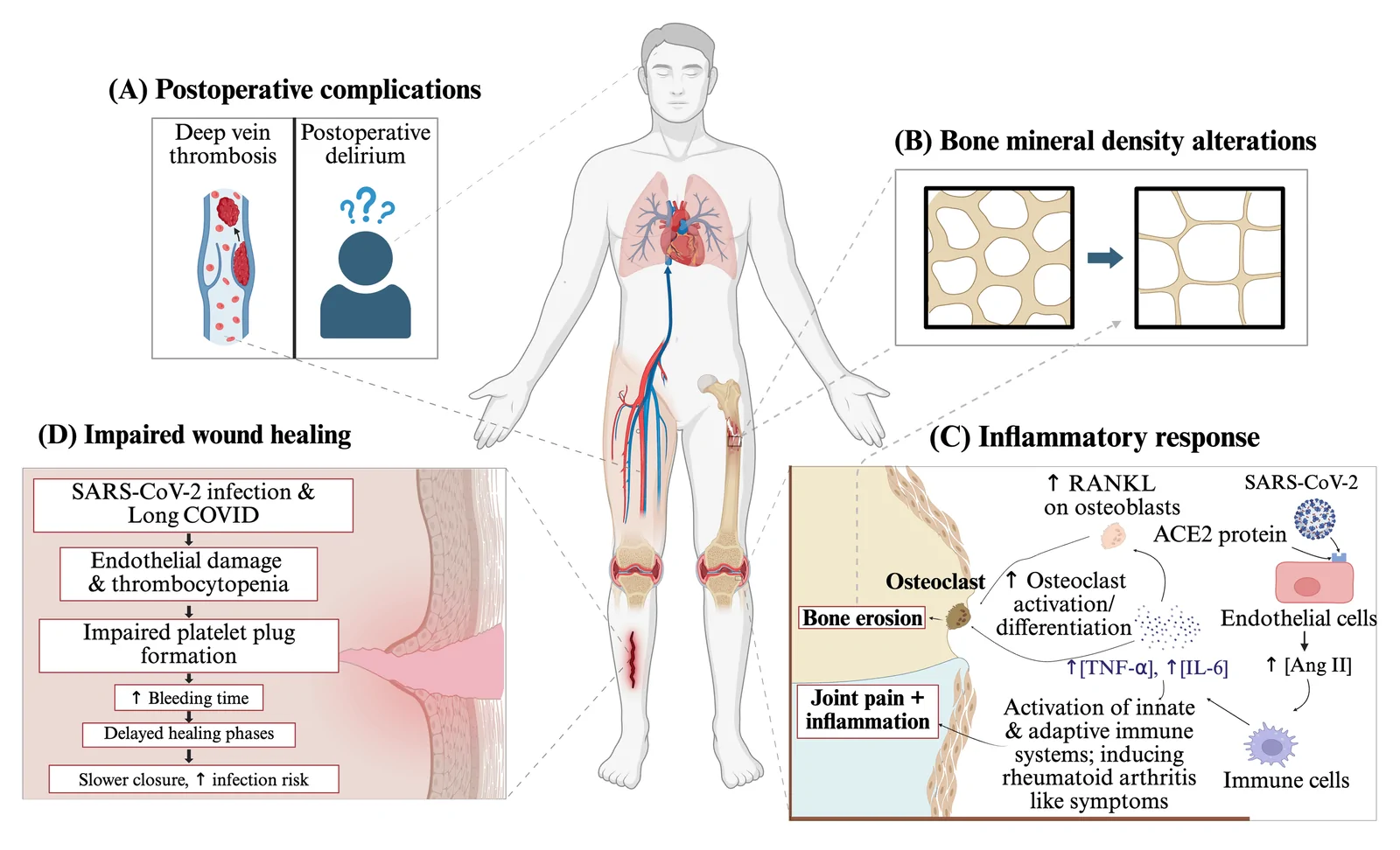
Orthopedic risks and adverse outcomes associated with Long COVID (A) Postoperative complications, including deep vein thrombosis and postoperative delirium. (B) Bone mineral density alterations due to dysregulated bone remodeling. (C) Pro-inflammatory state with increased TNF-α, IL-6, angiotensin II, and RANKL-mediated osteoclast activation, contributing to bone erosion and joint pain/inflammation. (D) Wound complications from impaired platelet plug formation, prolonged bleeding time, delayed healing, and increased risk of infection. Abbreviations: ACE2, angiotensin-converting enzyme 2; Ang II, angiotensin II; IL-6, interleukin-6; RANKL, receptor activator of nuclear factor κB ligand; TNF-α, tumor necrosis factor alpha. Created in BioRender. Zelenski, K. (2026). Sources: [9-15]

Bone mineral density alterations

The pathogenesis of COVID-19 begins with the cellular invasion of SARS-CoV-2 via angiotensin-converting enzyme 2 (ACE2) receptors, which are expressed in multiple tissues, including bone [10]. Viral-mediated downregulation of ACE2 increases angiotensin II levels, promoting systemic inflammation. This inflammatory reaction enhances osteoclast differentiation and increases osteoblast receptor activator of nuclear factor κB ligand expression, accelerating bone resorption (Figure 1B, 1C). Direct viral effects within the bone microenvironment have also been proposed. Gao et al. [16] demonstrated that SARS-CoV-2 infects bone marrow-derived macrophages, which are osteoclast precursors, via neuropilin-1 rather than ACE2, and that authentic viral infection impairs bone marrow-derived macrophage-to-osteoclast differentiation. These findings suggest dysregulation of osteoclastogenesis in the bone microenvironment. In parallel, another cause of decreased BMD is hypoxia [17]. COVID-19-associated hypoxia activates hypoxia-inducible pathways that promote bone resorption [18], while the systemic inflammatory response and endogenous glucocorticoid release further accelerate bone loss [19,20].

Clinically, reduced BMD in patients with Long COVID may predispose them to adverse outcomes. In a cohort of hospitalized COVID-19 patients, Wang et al. [11] reported a decline in BMD across all baseline categories, with the greatest loss observed among those with high baseline BMD. Notably, despite greater BMD loss in other groups, mortality rates were higher in the low-normal decline group (14.4% vs 8.0%), with lower odds of death in the high-rapid compared with the low-normal decline group (OR 0.52, 95% CI 0.32-0.83) [11]. This finding, along with a higher proportion with prolonged hospitalization (55.2% vs 42.9% hospitalized >10 days), suggests that pre-existing low baseline BMD, reflecting underlying frailty, may be a more powerful prognostic indicator than the absolute magnitude of acute BMD loss [11].

Similarly, di Filippo et al. [21] identified an association between Long COVID and vertebral fractures, showing that those with vertebral fractures were more likely to have Long COVID. In a cohort of 162 hospitalized COVID-19 survivors, morphometric vertebral fractures were incidentally detected on admission lateral chest radiographs in 25.9% of patients (54.8% moderate and 7.1% severe by Genant criteria). After 1:1 matching for age, sex, comorbidities, and acute disease severity, vertebral fractures emerged as the only independent risk factor for subsequent Long COVID (28.6% vs 9.5%, p = 0.04) [21]. Among patients with Long COVID, those with vertebral fractures had a higher prevalence of constitutional symptoms, including asthenia, myalgia, and arthralgia, compared with those without fractures (75% vs 31%, p = 0.04) [21].

These findings suggest that pre-existing skeletal fragility may preferentially predispose patients to the MSK symptom cluster most relevant to orthopedic recovery. Extending these observations, a cross-sectional study of 388 adults aged ≥55 years in Beijing reported a markedly higher prevalence of osteoporosis among individuals with SARS-CoV-2 reinfection compared with those infected only once (48.3% vs 17.3%, p < 0.0001), with consistent differences across the hip and lumbar spine; reinfection remained independently associated with osteoporosis after multivariable adjustment (OR 3.08, 95% CI 1.696-5.593) [22]. This association reinforces the view that COVID-related skeletal injury may compound with repeated exposure, a consideration of growing importance as the population accrues multiple lifetime SARS-CoV-2 infections. For orthopedic surgeons, these findings raise concern for compromised skeletal integrity in selected patients with Long COVID and support careful consideration of bone quality to mitigate postoperative complications, delayed union, or hardware failure.

Wound healing impairments and avascular necrosis

Wound healing is a tightly regulated cellular process involving sequential phases of hemostasis, inflammation, proliferation, and remodeling. COVID-19 may disrupt this physiological healing process through several mechanisms (Figure 1D) [23]. COVID-19-associated thrombocytopenia, reported in ~5-40% of cases, may impair platelet adhesion and prolong bleeding times, potentially compromising the early hemostatic phase [24]. In addition, SARS-CoV-2 infection has been associated with increased reactive oxygen species and inflammatory cytokine production, which may contribute to dysregulated inflammation and direct tissue injury [25]. However, the extent to which these mechanisms translate into clinically significant impairments in postoperative wound healing remains incompletely defined.

AVN has emerged as a notable MSK complication following SARS-CoV-2 infection. Several studies have suggested that osteonecrosis may occur earlier or more frequently following COVID-19 than in historical populations, although the available evidence is predominantly observational [14]. Endothelial dysfunction has been proposed as one potential mechanism linking SARS-CoV-2 infection to impaired bone perfusion. Viral infection and altered ACE2 signaling may disrupt vascular homeostasis, while endothelial injury, inflammation, and thrombosis could reduce regional blood flow and increase susceptibility to osteonecrosis [26]. Supporting this hypothesis, Kuliyeva et al. [27] identified distinctive mast cell clusters, fibrosis, and vascular thrombosis patterns in femoral head specimens from patients with prior COVID-19 compared with non-COVID arthroplasty patients. These findings provide mechanistic evidence for a potential vascular contribution to post-COVID osteonecrosis, although they do not establish SARS-CoV-2 infection as an independent causal factor. 

Treatment-related factors represent a separate and potentially better-established contributor to AVN risk in patients with severe COVID-19. Systemic glucocorticoids are an established risk factor for osteonecrosis and have become widely used in the treatment of severe COVID-19 [28,29]. Glucocorticoid exposure may disrupt bone and lipid metabolism, alter intraosseous pressure, and impair local blood flow, contributing to osteonecrosis [30]. Severe and multifocal AVN has been documented following corticosteroid treatment for COVID-19, including involvement of the femoral head and humerus [31]. A 2026 prospective cohort analysis using protocolized screening magnetic resonance imaging (MRI) in male survivors of severe COVID-19 who received high-dose corticosteroids reported a substantial prevalence of femoral head osteonecrosis [32]. While these findings strengthen the observed association between severe COVID-19, corticosteroid exposure, and subsequent osteonecrosis, the absence of an untreated comparison group limits causal inference.

More recent evidence further supports a multifactorial relationship between COVID-19, corticosteroid exposure, and AVN. A 2026 systematic review of 15 observational studies and case series found that corticosteroid exposure was common among patients who developed post-COVID femoral head AVN and was associated with greater disease severity, particularly with higher cumulative steroid doses [33]. However, AVN was also reported in several patients without corticosteroid exposure. Although these findings raise the possibility that COVID-19-related factors such as endothelial dysfunction, hypercoagulability, inflammation, or hypoxia may contribute independently to osteonecrosis, the available uncontrolled data cannot distinguish a direct effect of SARS-CoV-2 from unrecognized risk factors, pre-existing asymptomatic disease, or other confounding variables [33]. Thus, current evidence supports an association between COVID-19 and AVN rather than a causal relationship and suggests that post-COVID osteonecrosis is likely multifactorial, with corticosteroid exposure representing an important but not exclusive contributor [33].

Elevated inflammatory response and chronic pain

The sustained pro-inflammatory state associated with Long COVID is characterized by persistently elevated levels of pro-inflammatory cytokines, including interleukin-6 (IL-6), tumor necrosis factor-alpha, and interferon-gamma, which may persist for months after acute infection [6,9,26]. A 2026 transcriptomic analysis of two independent Long COVID cohorts confirmed these findings at a mechanistic level, demonstrating ongoing immune activation more than 180 days after infection. This was characterized by upregulation of Janus kinase-signal transducer and activator of transcription (JAK-STAT), IL-6, complement, and metabolic pathways, along with evidence of T-cell exhaustion [34]. Chronic cytokine release has been linked to neuroinflammation, which can alter pain processing and promote central sensitization, features that overlap with nociplastic pain, or pain without a specific cause (Figure 1C) [35]. Clinically, this may manifest as allodynia or hyperalgesia. Quantitative sensory testing provides objective evidence of dysregulated pain processing in Long COVID. Relative to healthy controls, patients with Long COVID demonstrate reduced pain detection and tolerance thresholds [36]. In a 2026 cohort of 84 individuals with post-COVID condition and 50 symptom-free controls, Lange et al. [36] demonstrated increased temporal summation of pain and a predominance of facilitatory, rather than inhibitory, conditioned pain modulation responses. These alterations were most pronounced in patients with chronic pain or more severe symptomatology and are consistent with central sensitization and a nociplastic pain phenotype [36].

Pain is among the most common and persistent manifestations of COVID-19 infection [37]. Oguz-Akarsu et al. [38] reported that approximately 72% of patients with mild-to-moderate COVID-19 experienced pain during the acute phase, and 31.9% continued to report persistent pain at 1.5-year follow-up [39]. Similarly, a 2025 systematic review and meta-analysis of 64 studies reported pooled prevalences of muscle pain, joint pain, and muscle weakness of 28%, 14.8%, and 12.9%, respectively, supporting the view that persistent MSK symptoms are a common component of Long COVID, although heterogeneity across studies was substantial [9].

The pathophysiology of chronic pain in Long COVID remains under investigation but appears to involve both inflammatory and autoimmune processes [40]. A case-control study reported elevated levels of inflammatory markers, including high-sensitivity C-reactive protein, in affected patients [41]. Microneurographic evidence suggests a peripheral substrate for painful Long COVID. In a retrospective cohort of patients with chronic post-COVID neuropathic symptoms, electrophysiological abnormalities in peripheral C fibers were identified in approximately 89% of those evaluated, indicating that small-fiber neuropathy may complement central sensitization as a key driver of persistent pain in this population [42].

Additional post-viral factors may further contribute, as patients with Long COVID exhibit increased rates of hypomagnesemia and 25-hydroxyvitamin D deficiency, both of which are associated with MSK pain and myalgia [43]. Collectively, these findings suggest that persistent inflammation, neuroimmune dysregulation, and micronutrient deficiencies may synergistically drive chronic pain in Long COVID.

Perioperative risk and postoperative complications

The persistent, multisystem effects of COVID-19 increase perioperative risks, particularly in patients with pre-existing comorbidities [44]. A growing body of literature demonstrates a continued heightened prevalence of thrombotic complications, including venous thromboembolism (VTE), across both the acute and post-acute phases of SARS-CoV-2 infection (Figure 1A) [45]. Although the precise mechanisms remain incompletely defined, persistent inflammation and chronic hypoxia are thought to induce endothelial dysfunction, thereby promoting a prothrombotic state [45,46]. A recent multinational study further corroborates these findings, demonstrating accelerated vascular aging after prior COVID-19 infection [47], thereby compounding age-related venous and endothelial changes recognized as contributors to VTE risk [45]. In the CARTESIAN cohort of 2,390 adults, adjusted carotid-femoral pulse wave velocity was approximately 0.4 m/s higher across all post-COVID groups, with the greatest increase (+1.09 m/s) observed in women requiring intensive care. This magnitude corresponds to an estimated 7.5 additional years of vascular aging and a 5.5% increase in projected cardiovascular event risk [47]. Given that orthopedic patient populations disproportionately include older women undergoing arthroplasty and fragility fracture care [48,49], this sex-specific amplification of vascular aging may have important implications for perioperative thromboembolic risk stratification [47].

Recent perioperative-focused reviews further emphasize that Long COVID is associated with persistent endothelial dysfunction and vasculopathy involving the pulmonary, cardiac, and peripheral vasculature, along with ongoing platelet activation and thromboembolic disease, which may complicate anesthetic planning [12]. Building on these observations, a systematic review reported sustained endothelial injury, immunothrombosis, microthrombosis, elevated D-dimer levels, and an increased risk of venous and arterial thrombotic events persisting for up to 49 weeks after the initial infection [50]. Beyond thrombotic risk, a nationwide matched cohort study of patients undergoing spinal surgery demonstrated that prior COVID-19 infection was associated with an approximately two-fold increase in the risk of spinal and implant-related infection within three months postoperatively on multivariable analysis, with elevated risk persisting when surgery was performed one to six months after infection [51]. These findings provide direct orthopedic evidence that post-COVID risk extends well beyond the acute phase and support vigilant infection control and wound management strategies in patients with a recent COVID-19 history undergoing instrumented procedures.

COVID-19 has also been associated with neurocognitive sequelae. Long COVID is linked to impairments in working memory, attention, and processing speed, commonly referred to as “brain fog,” and may worsen known risk factors for postoperative delirium [52-57]. Notably, independent of a perioperative setting, patients with Long COVID exhibit measurable deficits in executive function [58]. In a systematic review of 36 studies encompassing 3,256 adult survivors, Panagea et al. [58] reported a severity-graded prevalence of post-COVID cognitive impairment (29% in severe and 30% in moderate acute COVID-19 versus 3% in mild disease). The most consistently affected cognitive functions were executive function, attention, processing speed, and verbal and delayed-recall memory, with impairment rates varying across tests and cohorts. Intensive care unit admission, mechanical ventilation, severe pulmonary dysfunction, and lower cognitive reserve emerged as principal risk factors, several of which overlap with higher-acuity orthopedic perioperative populations [58]. In this context, it is concerning that, in a prospective cohort of older adults undergoing surgery, a history of COVID-19 was independently associated with an increased risk of postoperative delirium [59]. Postoperative delirium is already a common complication in older adults undergoing surgery, with reported incidence reaching up to 50% [60]. This is especially relevant in orthopedics, where prior reports indicate that more than 50% of procedures are performed on patients aged 65 or older [61]. Taken together, persistent Long COVID-related deficits in processing speed and executive function may further diminish perioperative cognitive reserve and heighten vulnerability to delirium.

Clinical implications for orthopedic practice

A core responsibility of the orthopedic surgeon is to individualize care by weighing the risks and benefits of intervention within the context of a patient’s broader medical status. Preoperative risk-stratification tools are increasingly incorporated into orthopedic decision-making, but these models rely on predefined variables and may not capture all clinically relevant sources of vulnerability [62]. Long COVID may therefore represent an underrecognized perioperative risk modifier that is not adequately reflected in current risk-stratification frameworks. With the WHO reporting more than 770 million cumulative SARS-CoV-2 infections worldwide to date, the MSK and orthopedic burden of Long COVID may be substantially greater than the current literature reflects [63]. This is clinically relevant, as the pathological states associated with Long COVID may manifest in domains already established as predictors of poor orthopedic outcomes, irrespective of COVID-19 etiology [64-69].

Translating this recognition into practice requires careful preoperative evaluation. Despite often normal laboratory and imaging findings, patients with Long COVID may exhibit reduced physiologic reserve [12,70]. A targeted history that assesses post-exertional malaise (PEM), postural orthostatic tachycardia syndrome (POTS), mast cell activation syndrome, persistent dyspnea, and cognitive dysfunction may help identify perioperative vulnerability [12,70]. Functional capacity should be evaluated using tools such as the Duke Activity Status Index or metabolic equivalents, which may detect decline not reflected in traditional comorbidity measures [71]. Referral should be symptom-guided, including cardiology for unexplained cardiopulmonary symptoms, autonomic specialists for dysautonomia or POTS, and pulmonary evaluation for significant respiratory burden [12,70,72]. BMD screening may also be considered in at-risk patients, such as adults over 50, postmenopausal women, those with a prior fragility fracture, and those on chronic corticosteroid treatment [10,21,22,73].

Perioperative management may require adjustment, as Long COVID is associated with endothelial dysfunction, accelerated vascular aging [46,47], and persistent platelet activation [12,45,46], which may further increase the already elevated risk of VTE in major orthopedic surgery [45,74]. Multidisciplinary consensus guidance recommends postponing elective surgery for at least two weeks after SARS-CoV-2 infection, with individualized risk assessment between two and seven weeks for higher-risk patients or procedures, with consideration of further delay for persistent symptoms [75]. However, orthopedic-specific recommendations for surgical timing in patients with established Long COVID have yet to be established. Current guidance favors lung-protective ventilation, careful fluid management, and consideration of regional or total intravenous anesthesia when feasible [12,72], along with multimodal, opioid-sparing analgesia to reduce respiratory depression [72]. Persistent cognitive deficits in executive function and processing speed [57] may further increase perioperative cognitive risk, particularly in older adults with prior COVID-19 [59], supporting delirium-prevention strategies such as minimizing deliriogenic medications and using multicomponent geriatric protocols.

Long COVID also poses distinct challenges to postoperative rehabilitation. Appelman et al. [76] reported lower exercise capacity, increased PEM, and systemic metabolic disturbances among patients with Long COVID compared with controls. Adherence to a rehabilitation protocol with progressive loading and functional use of the operative limb is crucial for achieving optimal long-term surgical outcomes. In this context, standard time-based orthopedic rehabilitation protocols may be poorly suited to patients with Long COVID. A 2025 systematic review and meta-analysis estimated a pooled prevalence of PEM of 25% (95% CI 17-36%) among community-dwelling adults with post-COVID condition [77]. In addition, a direct muscle biopsy study showed that exertion above an individualized symptom threshold has been associated with acute mitochondrial dysfunction, fiber atrophy, and immune cell infiltration [76]. Multidisciplinary guidance from the American Academy of Physical Medicine and Rehabilitation accordingly cautions that physical activity prescriptions must be carefully individualized to current tolerance, as excessive exertion may precipitate PEM and worsen functional status [70].

Structured pacing programs offer a potential alternative approach. The prospective PACELOC cohort reported a 15% weekly reduction in PEM episodes (95% CI 11-20%, p < 0.001) using an eight-week WHO Borg CR-10-guided protocol of incremental, symptom-bounded activity [78]. For orthopedic surgeons and physical therapists, the practical implication is that progression should be guided primarily by physiologic response to load rather than elapsed postoperative time, and that early co-management with physiatry or a Long COVID clinic may help optimize rehabilitation and reduce symptom exacerbations.

Beyond the initial orthopedic encounter, Long COVID may also affect long-term MSK health. A retrospective cohort study of patients aged ≥50 years across 141 healthcare organizations in North America and Western Europe reported an increased risk of new-onset osteoporosis following SARS-CoV-2 infection, with the highest observed risk occurring several years after infection and remaining elevated through six years of follow-up among individuals who developed new-onset overweight, obesity, or type 2 diabetes [73]. This finding complements prior observations that morphometric vertebral fractures during the index hospitalization were independently associated with subsequent Long COVID [21]. Together, these data suggest a potential for prolonged skeletal vulnerability in selected post-COVID populations, particularly those exposed to prolonged systemic glucocorticoid therapy during acute illness, and may support consideration of earlier bone density assessment in high-risk patients.

At present, however, evidence remains insufficient to incorporate Long COVID into formal orthopedic risk prediction models, and society-level orthopedic guidance is lacking. Informed consent in this population should therefore reflect residual uncertainty and evolving evidence, while incorporating multidisciplinary referral pathways where available to address fatigue, dysautonomia, mental health, and rehabilitation needs beyond the orthopedic episode of care [70]. Given an estimated global Long COVID prevalence of approximately 36% among individuals with confirmed SARS-CoV-2 infection [7], the downstream MSK burden may be substantial.

## Conclusions

This review highlights Long COVID as an emerging and underrecognized modifier of orthopedic risk, with potential implications for bone health, wound healing, pain, perioperative complications, and postoperative recovery. Given the global scale of SARS-CoV-2 exposure, even modest increases in MSK risk may translate into substantial clinical burden. The lack of guidance from orthopedic societies on managing patients with Long COVID represents a critical gap. This gap reflects an evidence base drawn largely from retrospective studies of non-orthopedic populations, difficulty separating post-viral pathophysiology from systemic corticosteroid effects, pre-existing frailty, and the multisystem nature of the condition. Clinicians are therefore left to extrapolate from multidisciplinary perioperative and rehabilitation frameworks developed for broader surgical populations. Future research should prioritize prospective, orthopedic-specific studies to define the prevalence, mechanisms, and outcome implications of Long COVID and to develop evidence-based perioperative and rehabilitation guidelines. Recognizing COVID-19 not as a resolved event but as a persistent determinant of MSK health will be essential for optimizing patient outcomes and strengthening preparedness for future public health crises.
